# MMP-9 upregulation may predict hemorrhagic transformation after endovascular thrombectomy

**DOI:** 10.3389/fneur.2024.1400270

**Published:** 2024-05-09

**Authors:** Jin-An Huang, Yu-Hsuan Wu, Po-Lin Chen, Yi-Chinn Weng, I-Chen Chiang, Yu-Ting Huang, Wen-Hai Chou

**Affiliations:** ^1^Department of Neurology, Neurological Institute Taichung Veterans General Hospital, Taichung, Taiwan; ^2^Department of Health Business Administration, Hungkuang University, Taichung, Taiwan; ^3^Department of Post-Baccalaureate Medicine, College of Medicine, National Chung Hsing University, Taichung, Taiwan; ^4^School of Medicine, Institute of Brain Science, National Yang Ming Chiao Tung University, Taipei, Taiwan; ^5^Center for Neuropsychiatric Research, National Health Research Institutes, Miaoli, Taiwan

**Keywords:** stroke, endovascular thrombectomy, biomarker, arterial blood, MMP-9, hemorrhagic transformation, personalized medicine

## Abstract

**Background:**

Hemorrhagic transformation (HT) is a serious complication after endovascular thrombectomy (EVT) for patients with acute ischemic stroke (AIS). We analyzed the plasma levels of MMP-9 before and after EVT and assessed the temporal changes of MMP-9 that may be associated with, and therefore predict, HT after EVT.

**Methods:**

We enrolled 30 AIS patients who received EVT, and 16 (53.3%) developed HT. The levels of MMP-9 in plasma collected from the arteries of AIS patients before and immediately after EVT were measured using ELISA. The percent change in MMP-9 after EVT (after/before) was calculated and compared between patients with and without HT.

**Results:**

The median age of the AIS patients was 70 years, and 13 patients (43.3%) were men. The median National Institutes of Health Stroke Scale (NIHSS) scores of patients with HT were 18 on admission and 18 after EVT. The median NIHSS scores of patients without HT were 17 on admission and 11 after EVT. Patients with HT demonstrated significantly greater percentage increases in arterial MMP-9 levels after EVT.

**Conclusion:**

Patients with AIS who developed HT had significantly increased arterial MMP-9 levels after EVT, suggesting that the upregulation of MMP-9 following EVT could serve as a predictive biomarker for HT.

## 1 Introduction

Ischemic stroke is the most prevalent neurological disease worldwide (1). Intravenous administration of recombinant tissue plasminogen activator (rt-PA) and endovascular thrombectomy (EVT) are the only two United States Food and Drug Administration (FDA)-approved reperfusion therapies for treating patients with acute ischemic stroke (AIS) (2). However, the therapeutic benefits of administering rt-PA 4.5 h after the onset of a stroke are overshadowed by the risks of hemorrhagic transformation (HT) (3). EVT, a more recently FDA-approved procedure, can be carried out within 24 h of the manifestation of symptoms and is primarily intended for AIS caused by large-vessel occlusions (4). Importantly, EVT can only be performed in stroke centers equipped with adequate resources and expertise (5). These constraints contribute to the estimation that less than 10% of stroke patients benefit from reperfusion therapies (6).

HT is a severe complication arising after reperfusion therapy that results in profound neurological impairment and significant mortality risk (3). Our clinical objective is to enhance early diagnosis, diminish the likelihood of cerebral hemorrhage, and ultimately augment the overall effectiveness of reperfusion therapies (7, 8). Matrix metalloproteinase-9 (MMP-9) belongs to the matrix metalloproteinase family and plays a role in proteolyzing cerebrovascular basement membrane and tight junction proteins (9, 10). Elevated expression of MMP-9 has been associated with breakdown of the blood–brain barrier (BBB) and development of HT following rt-PA treatments (11). However, these study results were acquired prior to the widespread adoption of EVT. One recent study determined the levels of serum biomarkers (MMP-9, APP770, ET-1, S100B, and Claudin-5) of venous blood collected from AIS patients at admission, yet predictive value of these biomarkers for HT following EVT was not demonstrated (12). The inability of serum biomarkers collected before EVT to predict HT could stem from factors intrinsic to the EVT procedure. During mechanical thrombectomy, direct vessel wall damage from endovascular procedures may exceed the BBB impairment resulting from ischemia (13). After three stent retriever passes during EVT, the rate of symptomatic intracranial hemorrhage or parenchymal hematoma increased exponentially post-puncture (14). Therefore, instead of relying on venous blood, we specifically collected arterial blood from the EVT puncture site to mitigate the influence of systemic factors. The arterial levels of MMP-9 collected both before and after EVT were analyzed to consider factors inherent in the EVT process. Our findings indicate that the increased levels of arterial MMP-9 post-EVT were associated with incidence of HT and could potentially function as a predictive biomarker for HT following EVT.

## 2 Materials and methods

### 2.1 Study design and subjects

This was a single-center, prospective cohort study. Stroke patients older than 20 years who underwent EVT at Taichung Veterans General Hospital (TCVGH) were enrolled between July 2022 and April 2023. Cerebral ischemia with large-vessel occlusion (LVO) was diagnosed by a neurologist and supported by Computed Tomography (CT) perfusion scan. This study protocol was approved by the Institutional Review Board (IRB) of TCVGH (CE22262B) and by National Health Research Institutes (NHRI, EC1110609-E). Written informed consent was obtained from the patients or their legally authorized representatives.

### 2.2 Data collection

The clinicodemographic data collected included age, gender, diagnoses of hypertension, diabetes mellitus, dyslipidemia, or atrial fibrillation, smoking history, previous transient ischemic attack (TIA) or stroke, prior congestive heart failure, prior rt-PA administration, levels of plasma glucose, HbA1c, fibrinogen, D-dimer, and low-density lipoprotein (LDL), as well as score on the National Institutes of Health Stroke Scale (NIHSS). The etiology of ischemic stroke was categorized based on the Trial of ORG 10172 in Acute Stroke Treatment (TOAST) criteria, distinguishing between large-artery atherosclerosis, cardioembolism, or other causes (15).

Occluded vessels were identified using brain Computed Tomography Angiography (CTA). Times from stroke onset to arterial blood sampling before therapy and after EVT were recorded. Successful reperfusion was determined by attaining a modified Treatment in Cerebral Infarction (mTICI) score of 2b, 2c, or 3. NIHSS was evaluated again 24 h after EVT. Dual-energy Computed Tomography (CT) scans were conducted 24 h after EVT to assess HT. Brain Magnetic Resonance Angiography (MRA) was performed in accordance with clinical necessity, determination of late-onset HT, and stroke location. The categorization of HT was determined in accordance with the Heidelberg criteria (16). All brain images were evaluated by radiologists who were blinded to the laboratory determinations and clinical outcomes.

The primary outcome of this study was HT within a week of admission. Malignant edema was defined as the presence of midline shift ≥5 mm. Functional independence, determined by the modified Rankin Scale (mRS) score, was indicated by scores ranging from 0 to 2.

### 2.3 Enzyme-linked immunosorbent assay

Arterial blood was collected from the puncture site, in a BD Vacutainer^®^ EDTA tube (Becton, Dickinson and Company, New Jersey, United States), before and after EVT and centrifuged at 1,500 × g for 15 min at 4°C. The supernatant was collected as blood plasma. The level of MMP-9 in the plasma was measured following the manufacturer’s protocol for the human MMP-9 Quantikine ELISA kit (R&D, Minneapolis, MN, United States).

### 2.4 Statistical analysis

Continuous variables were expressed as mean ± standard deviation (SD) or median (interquartile range, IQR) and compared using the Mann–Whitney U test. Categorical variables were expressed as percentages and compared using Fisher’s exact test. The plasma levels of MMP-9 before and after EVT were compared between HT and non-HT patients using one-way ANOVA with Newman–Keuls *post hoc* tests. The percentage change in MMP-9 level was calculated by dividing the MMP-9 level after EVT by the MMP-9 level before EVT; the changes observed among non-HT and HT patients were then compared using the two-tailed, unpaired *t*-test. The diagnostic potential of MMP-9 level change in predicting HT and the cut-off values was evaluated using the receiver operating characteristic (ROC) in simple logistic regression analysis. All data analyses were performed using Prism 9 (GraphPad, La Jolla, CA, United States). A statistically significant difference was defined as *p* < 0.05.

## 3 Results

### 3.1 Baseline characteristics of stroke patients

**Figure 1:**
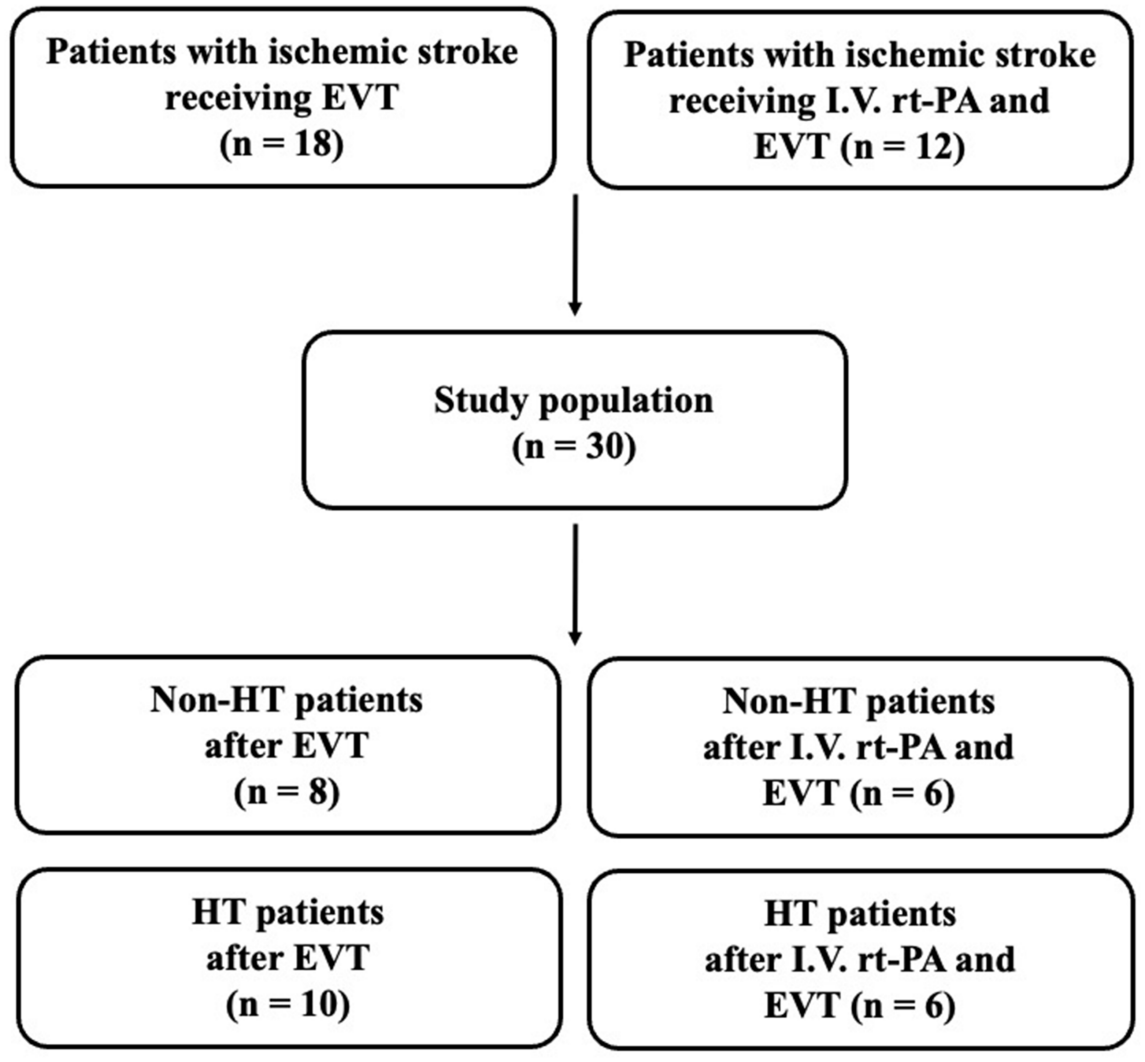
Flowchart of stroke patient inclusion. EVT, endovascular thrombectomy; I.V., intravenous; HT, hemorrhagic transformation; rt-PA, recombinant tissue plasminogen activator.

From July 2022 through April 2023, a total of 30 stroke patients received EVT at TCVGH and were enrolled in this study. Among the 30 patients, 18 stroke patients underwent EVT and 12 patients underwent both rt-PA and EVT therapy (Figure 1). Sixteen patients developed HT after EVT, while 14 did not experience HT (non-HT). Among the HT patients, we observed that 3 patients (18.75%) developed hemorrhagic infarction 1 (HI1), 2 patients (12.5%) developed HI2, 7 patients (43.75%) developed parenchymal hematoma 1 (PH1), 1 patient (6.25%) developed PH2, while 3 patients (18.75%) exhibited subarachnoid hemorrhage (SAH), subdural hematoma (SDH), or intraventricular hemorrhage (IVH) each. Furthermore, only 2 patients (12.5%) experienced delayed HT.

The baseline characteristics of patients are shown in Table 1. The median age was 70 years, and 43.3% were men. The most common type of occlusion was the insular segment of the middle cerebral artery (MCA; M2, 33.3%), followed by the horizontal segment of the MCA (M1, 30.0%), extracranial internal carotid artery (13.3%), posterior circulation (13.3%), and intracranial internal carotid artery (10.0%). The median NIHSS score upon admission was 18, which subsequently reduced to 14 within 24 h following reperfusion therapy and hospitalization. The median time between stroke onset and arterial blood sampling was 5.7 h before EVT and 6.6 h after EVT. There were no significant differences in demographics, medical history, laboratory findings at admission, or clinical characteristics between non-HT and HT patients. In the evaluation of clinical outcomes, it was found that non-HT patients showed significant improvement in functional independence upon discharge. There were trends toward improvement in the NIHSS 24 h after EVT and functional independence at the 3rd month in non-HT patients, but the differences were not significant.

**Table 1:** Baseline characteristics of patients with and without HT after EVT.

	All patients (*n* = 30)	Non-HT (*n* = 14)	HT (*n* = 16)	*p*-value
**Demographics**				
Age (years), median (IQR)	70 (61–76)	68 (57–77)	71 (65–76)	0.735
Male, *n* (%)	13 (43.3%)	7 (50.0%)	6 (37.5%)	0.713
**Medical history**				
Hypertension, *n* (%)	23 (76.7%)	12 (85.7%)	11 (68.8%)	0.399
Diabetes mellitus (DM), *n* (%)	7 (23.3%)	3 (21.4%)	4 (25.0%)	0.999
Dyslipidemia, *n* (%)	17 (56.7%)	8 (57.1%)	9 (56.3%)	0.999
Atrial fibrillation, *n* (%)	18 (60.0%)	9 (64.3%)	9 (56.3%)	0.722
Smoking, *n* (%)	5 (16.7%)	4 (28.6%)	1 (6.3%)	0.157
Previous TIA or stroke, *n* (%)	10 (33.3%)	4 (28.6%)	6 (37.5%)	0.709
Congestive heart failure, *n* (%)	4 (13.3%)	1 (7.1%)	3 (18.8%)	0.602
rt-PA administration, *n* (%)	12 (40.0%)	6 (42.9%)	6 (37.5%)	0.999
**Laboratory findings at admission**				
Plasma glucose, mg/dL	151.6 ± 67.4	156.2 ± 89.2	147.6 ± 43.0	0.454
HbA1c, mg/dL	6.4 ± 1.5	6.3 ± 1.4	6.5 ± 1.6	0.659
Fibrinogen, mg/dL	308.3 ± 136.9	301.7 ± 91.5	316.2 ± 182.7	0.807
D-dimer, mg/L FEU	4.6 ± 7.4	5.9 ± 10.3	3.5 ± 3.7	0.939
LDL, mg/dL	91.8 ± 29.5	95.8 ± 27.3	88.3 ± 31.8	0.394
**Clinical characteristics**				
NIHSS at admission, median (IQR)	18 (12–22)	17 (12–24)	18 (11–21)	0.894
**TOAST**				
Large artery atherosclerosis, *n* (%)	6 (20.0%)	3 (21.4%)	3 (18.8%)	0.999
Cardioembolism, *n* (%)	20 (66.7%)	9 (64.3%)	11 (68.8%)	0.999
Other, *n* (%)	4 (13.3%)	2 (14.3%)	2 (12.5%)	0.999
**Occluded vessels**				
Extracranial internal carotid artery, *n* (%)	4 (13.3%)	1 (7.1%)	3 (18.8%)	0.602
Intracranial internal carotid artery, *n* (%)	3 (10%)	2 (14.3%)	1 (6.3%)	0.586
Horizontal segment of MCA (M1), *n* (%)	9 (30%)	3 (21.4%)	6 (37.5%)	0.440
Insular segment of MCA (M2), *n* (%)	10 (33%)	5 (35.7%)	5 (31.3%)	0.999
Posterior circulation, *n* (%)	4 (13.3%)	3 (21.4%)	1 (6.3%)	0.316
Time from stroke onset to arterial blood sampling before therapy, h	5.7 (4.0–8.8)	5.7 (4.1–16.7)	5.7 (3.5–7.4)	0.396
Time from stroke onset to EVT and arterial blood sampling after EVT, h	6.6 (4.8–9.7)	6.6 (4.8–17.8)	6.8 (4.7–9.3)	0.520
Successful reperfusion, *n* (%)^**#**^	22 (73.3%)	12 (85.7%)	10 (62.5%)	0.226
**Clinical outcome**				
Malignant edema, *n* (%)	3 (10%)	0 (0%)	3 (18.8%)	0.228
NIHSS 24 h later, median (IQR)	14 (8–21)	11 (8–16)	18 (9–22)	0.119
Functional independence at discharge	4 (13.3%)	4 (28.6%)	0 (0%)	0.037^*^
Functional independence at 3rd month^**##**^	9 (30%)	6 (42.9%)	3 (18.8%)	0.236

HT, hemorrhagic transformation; EVT, endovascular thrombectomy; TIA, transient ischemic attack; rt-PA, recombinant tissue plasminogen activator; mg/dL, milligram/deciliter; HbA1c, glycated hemoglobin; LDL, low density lipoproteins; NIHSS, National Institutes of Health Stroke Scale; TOAST, Trial of Org 10172 in Acute Stroke Treatment; MCA, middle cerebral artery.

*p < 0.05. ^#^Successful reperfusion was defined as achieving a modified treatment in cerebral infarction (mTICI) score of 2b, 2c, or 3. ^##^Functional independence was defined as Modified Rankin scale (mRS) ranging from 0 to 2.

### 3.2 Upregulation of MMP-9 after EVT is associated with HT

**Figure 2:**
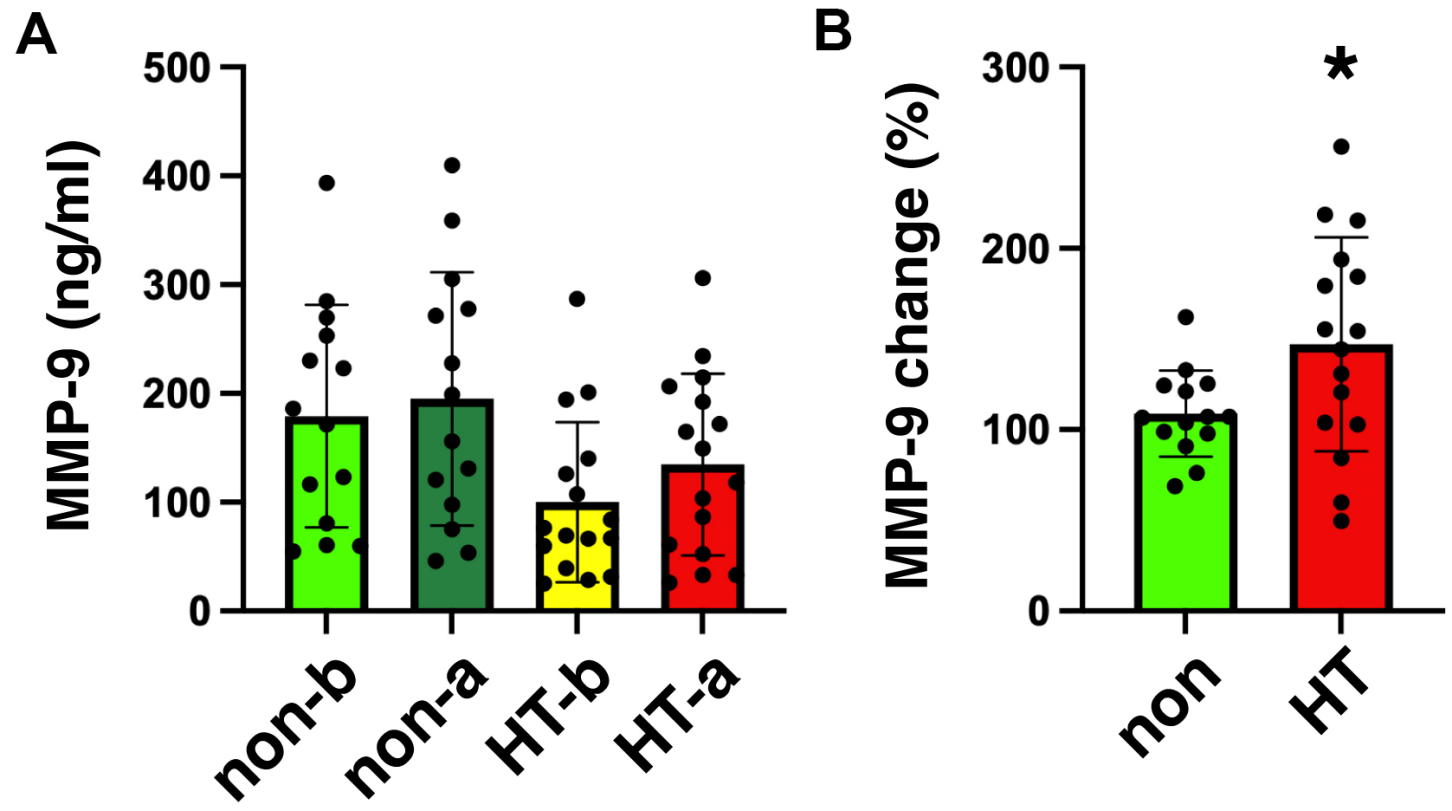
Plasma levels of MMP-9 in stroke patients collected before and after EVT. **(A)** Levels of MMP-9 in the plasma of stroke patients collected before and after EVT measured by ELISA. Data on MMP-9 concentration in plasma from non-HT patients collected before (non-b) and after EVT (non-a), as well as from HT patients collected before (HT-b) and after (HT-a) EVT were analyzed via one-way ANOVA with Newman–Keuls *post hoc* tests. **(B)** Percentage change in MMP-9 level (after/before) among non-HT and HT patients. There was a significant increase in percentage change of MMP-9 level after EVT in HT patients (two-tailed, unpaired *t*-test, *p < 0.05). EVT, endovascular thrombectomy; HT, hemorrhagic transformation; MMP-9, matrix metalloproteinase-9.

**Figure 3:**
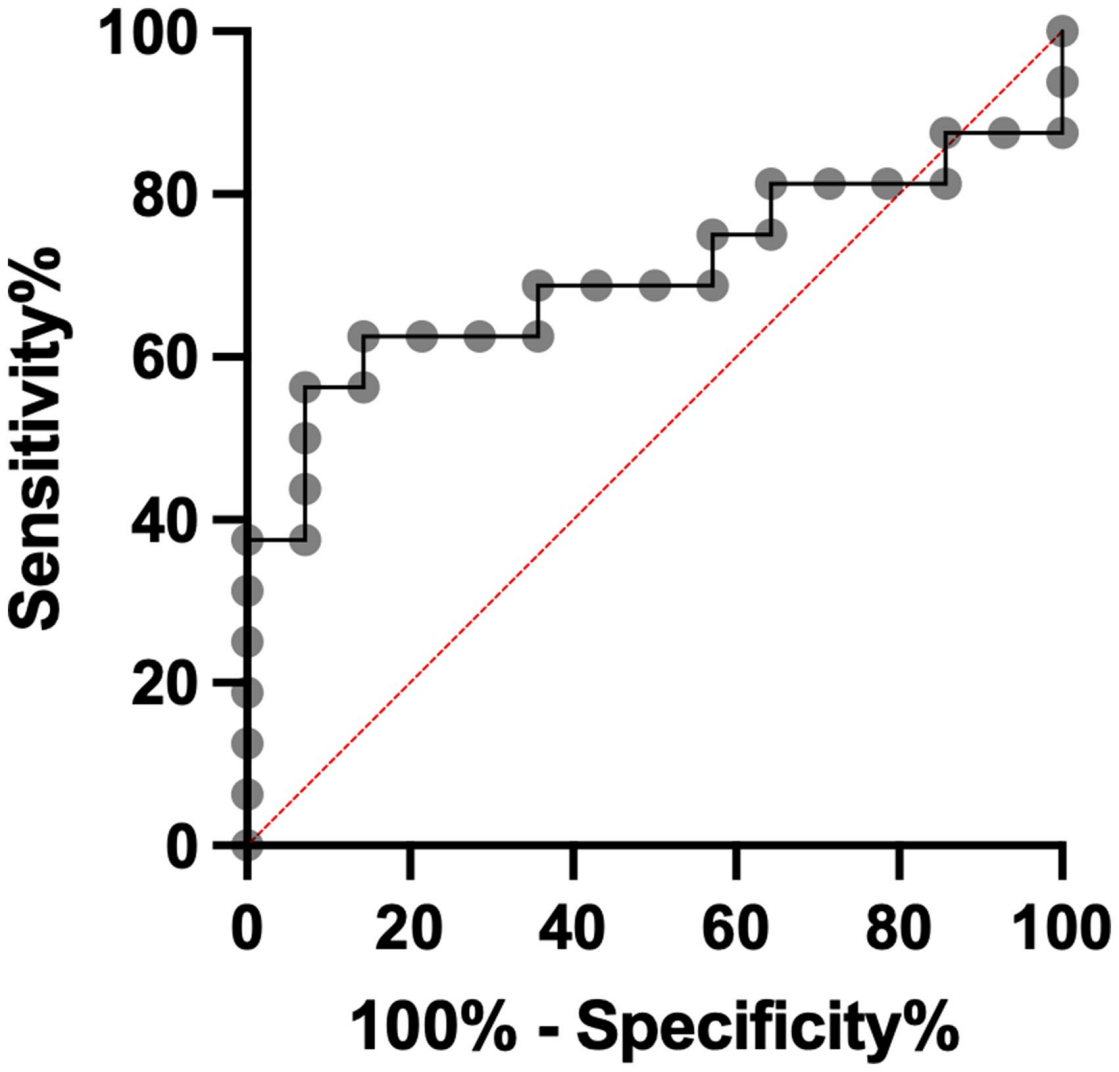
Specificity and sensitivity of utilization of plasma MMP-9 percentage change to diagnose HT after EVT. EVT, endovascular thrombectomy; HT, hemorrhagic transformation; MMP-9, matrix metalloproteinase-9.

The mean arterial MMP-9 level in non-HT patients was 179.1 ng/mL before EVT and increased to 195.0 ng/mL after EVT (Figure 2A). For HT patients, the mean arterial MMP-9 level was 100.1 ng/mL before EVT and increased to 134.6 ng/mL after EVT. Although the arterial MMP-9 levels were elevated after EVT in both non-HT and HT patients, these differences did not achieve statistical significance. We calculated the percentage change in arterial MMP-9 for individual patients before and after EVT, revealing a percentage change of 108.8% for non-HT and 147.1% for HT patients (Figure 2B). Interestingly, the percentage changes in arterial MMP-9 after EVT were significantly higher in HT patients compared to non-HT patients. An ROC curve was generated for the MMP-9 percentage changes, with an area under the curve (AUC, 95% confidence interval [CI]) value of 0.701 (Figure 3). The optimal cut-off value of plasma MMP-9 upregulation for the diagnosis of HT versus non-HT is 128.1% (sensitivity = 62.5%, specificity = 85.7%).

We have included supplemental figures showing the MMP-9 levels (Supplementary Figure S1A) and the MMP-9 percentage changes (Supplementary Figure S1B) of patients who received EVT treatment alone as well as patients who received both I.V. rt-PA and EVT. The arterial MMP-9 levels and changes were compared between these groups using one-way ANOVA and Tukey’s *post hoc* tests. No significant difference was found between the groups.

The arterial MMP-9 change for non-HT male is 111.9, 133.6% for HT males, 105.7% for non-HT females, and 155.2% for HT females (Supplementary Figure S2). The arterial MMP-9 changes of these four groups were compared using one-way ANOVA and Tukey’s *post hoc* tests. No significant difference was found between these groups.

## 4 Discussion

Our results demonstrate that the upregulation of arterial MMP-9 following EVT is associated with the occurrence of HT and may serve as a predictive biomarker for HT after EVT. To the best of our knowledge, this study represents the first research, to date, evaluating the prognostic role of temporal changes in arterial biomarkers after EVT.

Our main finding was partly in line with the large retrospective study where higher admission neutrophil-to-lymphocyte ratio (NLR) was an independent predictor of symptomatic intracranial hemorrhage (sICH) in AIS patients treated with EVT (17). AIS has been shown to trigger a strong immune system response in animal models of MCA occlusion by enhancing neutrophil migration into the brain. Neutrophils are a key source of MMP-9 within the first 24 h after AIS, potentially linking admission NLR to sICH in LVO patients post-EVT, given the association of MMP-9 with BBB disruption and sICH after thrombolysis (17). However, several factors can influence admission NLR, including infections, inflammatory conditions, stress, and certain medications (18).

Several studies indicated that higher levels of MMP-9 before thrombolytic therapies were associated with the development of HT after rt-PA administration (10). The median MMP-9 values detected in blood plasma before rt-PA treatments ranged from 54 to 225 ng/mL (11), a range akin to the MMP-9 levels (100.1–195 ng/mL) observed in our study utilizing blood plasma. A recent study revealed that high MMP-9 levels in blood sera (>775 ng/mL) at 6 h from admission were associated with HT following EVT (19). Conversely, another recent study utilizing blood sera failed to establish a connection between MMP-9 levels (15–46 ng/mL) and HT following EVT (12). MMP-9 levels vary significantly across studies, likely due to the utilization of different ELISA kits sourced from various companies and differences in sample types, such as serum versus plasma (20, 21). The processing of blood samples to obtain sera versus plasma can contribute to differences in MMP-9 levels. Serum is obtained by allowing blood to clot and then centrifuging to separate the liquid portion (serum) from the clot. Plasma, on the other hand, is obtained by centrifuging blood without allowing it to clot, resulting in a liquid portion containing clotting factors and cellular components. Neutrophils are known to be a significant source of MMP-9, and their activation and degranulation can lead to increased MMP-9 secretion (20, 21). During the clotting process in serum preparation, neutrophils may release MMP-9 into the serum, contributing to higher MMP-9 levels compared to plasma samples, where neutrophils are not activated (20, 21).

Personalized medicine, also known as precision medicine, represents an innovative paradigm in medical treatment and healthcare (22). Rather than adopting a one-size-fits-all approach, personalized medicine aims to tailor medical decisions, interventions, and treatments to the specific characteristics of each patient (23). In this study, we embraced the personalized medicine approach by examining the percentage changes in arterial MMP-9 levels before and after EVT in individual patients to identify those at high risk of HT. Although potential gender-related differences in MMP-9 levels in cardiovascular and central nervous system conditions have been reported (24), no significant gender difference of arterial MMP-9 changes was found in this study.

By concentrating resources on individuals at a heightened risk of HT resulting from these interventions, we aim to optimize the utilization of resources and improve patient outcomes. For example, several small-molecule inhibitors targeting MMP-9 have been developed (25). Recently, an anti-MMP-9 neutralizing antibody was isolated based on a functional selection strategy (26). This antibody demonstrated the ability to reduce BBB breakdown in mice following a stroke (27). Our study may assist in identifying and prioritizing patients who would derive the greatest benefits from MMP-9 inhibitors, aiming to prevent or minimize the occurrence of hemorrhagic complications (28).

A major limitation of our study was its relatively small sample size. Nonetheless, the upregulation of MMP-9 showed clear associations with the occurrence of HT following EVT. The application of personalized medicine supported by our study can be substantiated through broader and more extensive investigations. However, a limitation preventing practical clinical use of MMP-9 as a biomarker is the length of time required to obtain results from existing MMP-9 ELISA assays; the current assays require over 3 h to produce results. The development of more rapid MMP-9 assays is crucial for practical clinical application.

## 5 Conclusion

In conclusion, the results from this single-center prospective study demonstrate an association between MMP-9 upregulation following EVT and the occurrence of HT. We are the first to show that the upregulation of MMP-9 may act as a biomarker for HT following EVT. The idea of biomarker-guided therapy, as suggested in this study, has been explored and applied using the association between various other biomarkers and the clinical status of patients (29, 30). This approach may foster more personalized and targeted strategies in stroke care, ultimately enhancing patient outcomes.

## Data availability statement

The original contributions presented in the study are included in the article/Supplementary material, further inquiries can be directed to the corresponding author.

## Ethics statement

This study protocol was approved by the Institutional Review Board (IRB) of Taichung Veterans General Hospital (TCVGH, CE22262B) and by National Health Research Institutes (NHRI, EC1110609-E). The studies were conducted in accordance with the local legislation and institutional requirements. The participants provided their written informed consent to participate in this study. Written informed consent was obtained from the individual(s) for the publication of any potentially identifiable images or data included in this article.

## Author contributions

J-AH: Writing – review & editing, Writing – original draft, Supervision, Project administration, Investigation, Funding acquisition, Formal analysis, Data curation, Conceptualization. Y-HW: Writing – review & editing, Writing – original draft, Validation, Supervision, Project administration, Methodology, Investigation, Funding acquisition, Formal analysis, Data curation, Conceptualization. P-LC: Writing – review & editing, Writing – original draft, Supervision, Project administration, Methodology, Investigation, Formal analysis, Data curation, Conceptualization. Y-CW: Writing – review & editing, Visualization, Validation, Supervision, Software, Resources, Project administration, Methodology, Investigation, Funding acquisition, Formal analysis, Data curation, Conceptualization. I-CC: Writing – review & editing, Visualization, Validation, Supervision, Software, Resources, Project administration, Methodology, Investigation, Funding acquisition, Formal analysis, Data curation, Conceptualization. Y-TH: Writing – review & editing, Visualization, Validation, Supervision, Software, Resources, Project administration, Methodology, Investigation, Funding acquisition, Formal analysis, Data curation, Conceptualization. W-HC: Writing – review & editing, Writing – original draft, Visualization, Validation, Supervision, Software, Resources, Project administration, Methodology, Investigation, Funding acquisition, Formal analysis, Data curation, Conceptualization.

## Supplementary material

SUPPLEMENTARY FIGURE S1 Plasma levels of MMP-9 collected before and after EVT among stroke patients who received EVT alone and patients who received both rt-PA and EVT. **(A)** The MMP-9 levels are from non-HT patients collected before EVT (non-b-EVT), non-HT patients collected after EVT (non-a-EVT), non-HT patients collected after rt-PA and before EVT (non-b-rtPA+EVT), non-HT patients collected after rt-PA and EVT (non-a-rtPA+EVT), HT patients collected before EVT (HT-b-EVT), HT patients collected after EVT (HT-a-EVT), HT patients collected after rt-PA and before EVT (HT-b-rtPA+EVT), and HT patients collected after rt-PA and EVT (HT-a-rtPA+EVT). The plasma levels of MMP-9 were compared between different groups using one-way ANOVA with Newman–Keuls *post hoc* tests. No significant difference was found between these groups. **(B)** The MMP-9 percentage changes are from non-HT patients who received EVT alone (non-EVT), non-HT patients who received both rt-PA and EVT, HT patients who received EVT alone (HT-EVT), and HT patients who received both rt-PA and EVT (HT-rtPA+EVT). The percentage changes of MMP-9 were compared between different groups using one-way ANOVA with Newman–Keuls *post hoc* tests. No significant difference was found between these groups. EVT, endovascular thrombectomy; HT, hemorrhagic transformation; MMP-9, matrix metalloproteinase-9.

SUPPLEMENTARY FIGURE S2 Percentage changes in MMP-9 level (after/before) among male and female patients with ischemic stroke. The MMP-9 percentage changes in plasma from male non-HT patients (male-non), male HT patients (male-HT), female non-HT patients (female-non), and male HT patients (female-HT) were analyzed using one-way ANOVA with Newman–Keuls *post hoc* tests. No significant difference was found between these groups. HT, hemorrhagic transformation; MMP-9, matrix metalloproteinase-9.
