# Supplementary figures and images for: MMP-9 upregulation may predict hemorrhagic transformation after endovascular thrombectomy
Source: Front Neurol. 2024 May 9;15:1400270. doi: 10.3389/fneur.2024.1400270 (PMC11119322; doi:10.3389/fneur.2024.1400270)

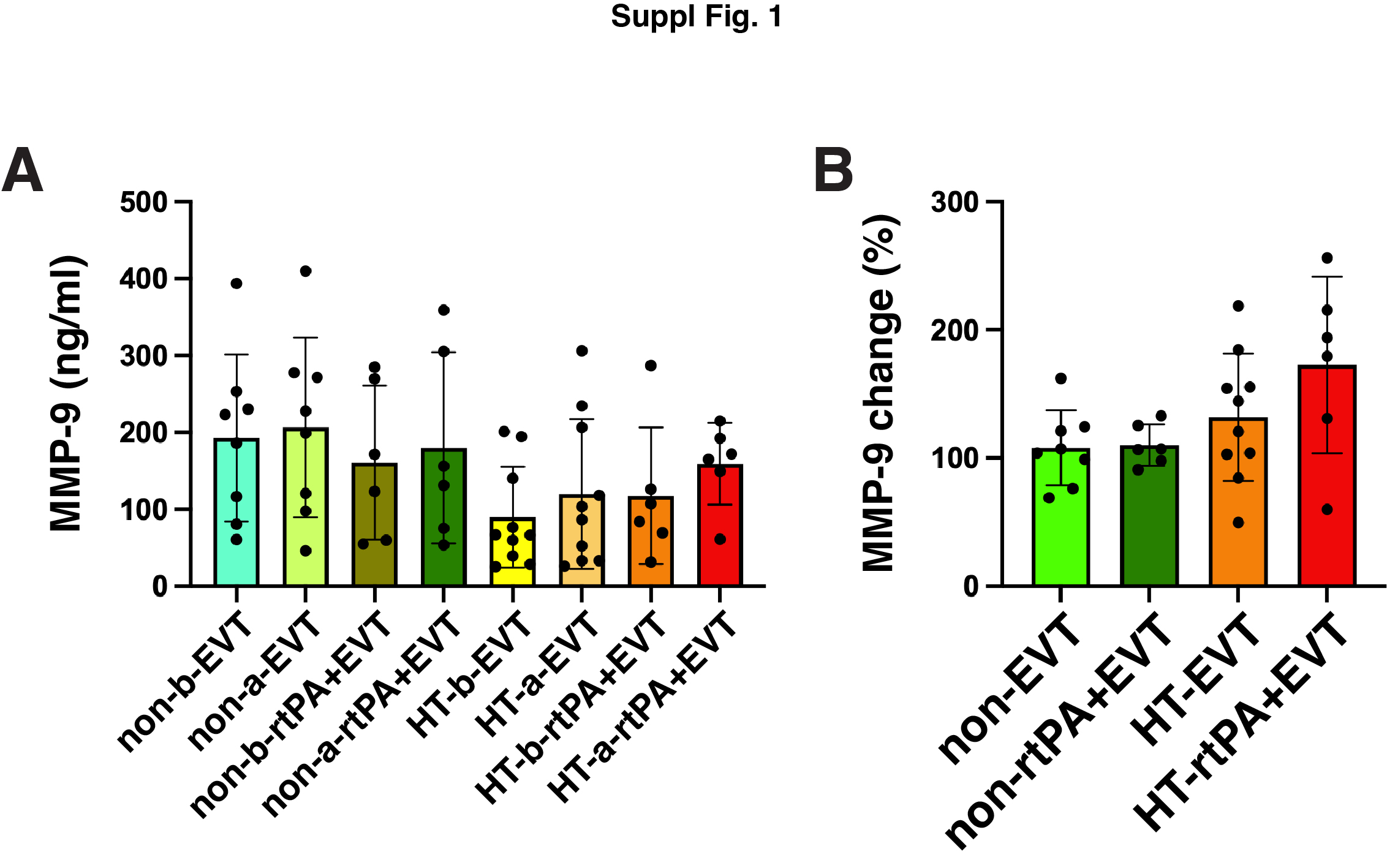

Supplement: SUPPLEMENTARY FIGURE S1 — Plasma levels of MMP-9 collected before and after EVT among stroke patients who received EVT alone and patients who received both rt-PA and EVT. (A) The MMP-9 levels are from non-HT patients collected before EVT (non-b-EVT), non-HT patients collected after EVT (non-a-EVT), non-HT patients collected after rt-PA and before EVT (non-b-rtPA+EVT), non-HT patients collected after rt-PA and EVT (non-a-rtPA+EVT), HT patients collected before EVT (HT-b-EVT), HT patients collected after EVT (HT-a-EVT), HT patients collected after rt-PA and before EVT (HT-b-rtPA+EVT), and HT patients collected after rt-PA and EVT (HT-a-rtPA+EVT). The plasma levels of MMP-9 were compared between different groups using one-way ANOVA with Newman–Keuls post hoc tests. No significant difference was found between these groups. (B) The MMP-9 percentage changes are from non-HT patients who received EVT alone (non-EVT), non-HT patients who received both rt-PA and EVT, HT patients who received EVT alone (HT-EVT), and HT patients who received both rt-PA and EVT (HT-rtPA+EVT). The percentage changes of MMP-9 were compared between different groups using one-way ANOVA with Newman–Keuls post hoc tests. No significant difference was found between these groups. EVT, endovascular thrombectomy; HT, hemorrhagic transformation; MMP-9, matrix metalloproteinase-9. [file Image_1.jpeg]

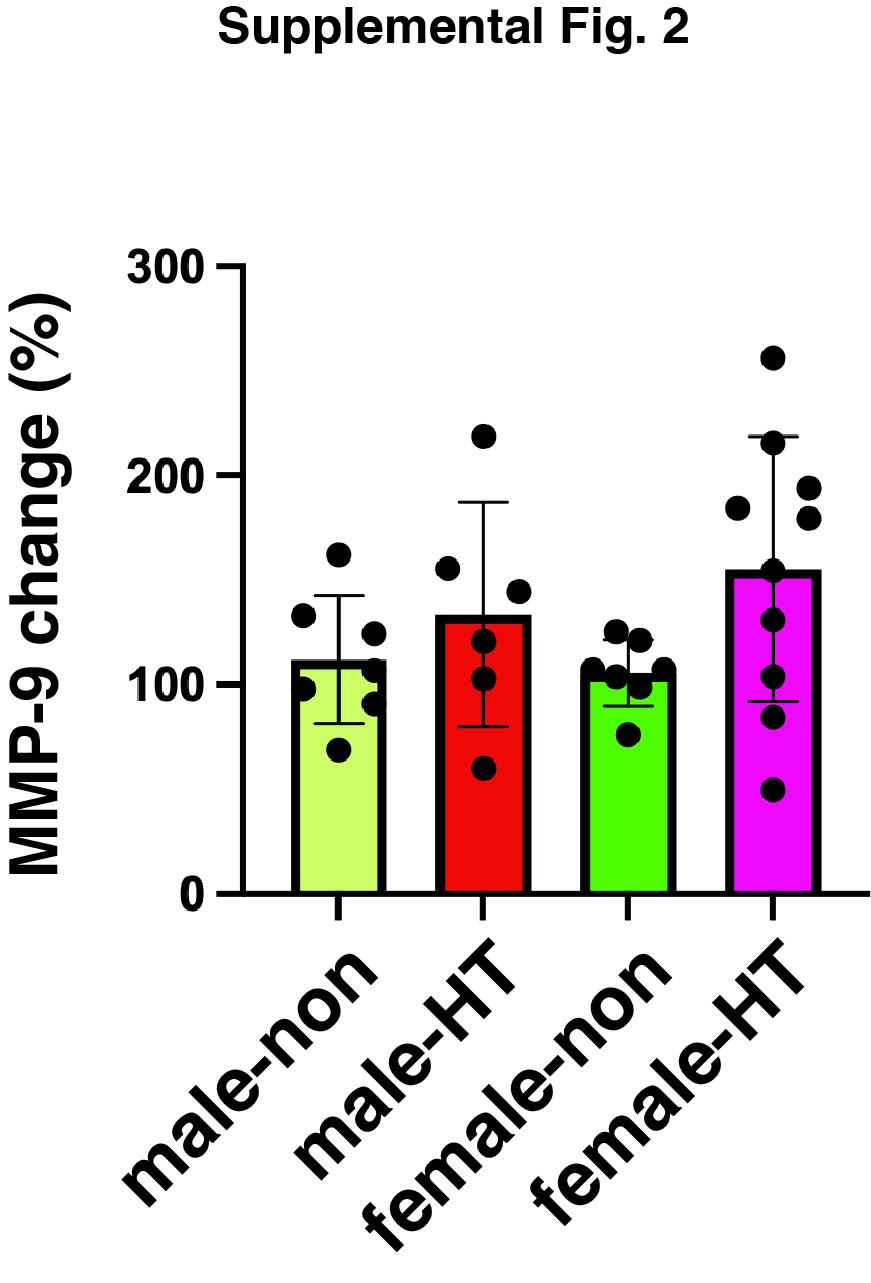

Supplement: SUPPLEMENTARY FIGURE S2 — Percentage changes in MMP-9 level (after/before) among male and female patients with ischemic stroke. The MMP-9 percentage changes in plasma from male non-HT patients (male-non), male HT patients (male-HT), female non-HT patients (female-non), and male HT patients (female-HT) were analyzed using one-way ANOVA with Newman–Keuls post hoc tests. No significant difference was found between these groups. HT, hemorrhagic transformation; MMP-9, matrix metalloproteinase-9. [file Image_2.jpeg]
